# Inflammatory Cytokines as Potential Inducements of Early Gastric Mucosal Lesions in *Helicobacter pylori*‐Infected Patients

**DOI:** 10.1155/grp/9063312

**Published:** 2026-05-18

**Authors:** Minghong Li, Chiyu Tian, Kai Guo, Wei Pan, Runhua Lv, Lipeng Jing, Dongmei Yang

**Affiliations:** ^1^ Department of Clinical Laboratory, Jing Yuan County Hospital of Traditional Chinese Medicine, Baiyin, China; ^2^ Institute of Epidemiology and Statistics, School of Public Health, Lanzhou University, Lanzhou, China, lzu.edu.cn; ^3^ Department of Cardiology and Cardiovascular Research Institute, Renmin Hospital of Wuhan University, Wuhan, Hubei, China, rmhospital.com; ^4^ Department of Public Health, Jing Yuan County Hospital of Traditional Chinese Medicine, Baiyin, China; ^5^ Jing Yuan County Hospital of Traditional Chinese Medicine, Baiyin, China

**Keywords:** ^13^C-urea breath test, *Helicobacter pylori*, inflammatory cytokines, pepsinogen, segmental regression

## Abstract

**Background:**

Chronic inflammation due to *Helicobacter pylori* infection causes the development of many gastrointestinal diseases, such as gastric ulcer and atrophic gastritis. This study is aimed at investigating the relationship between inflammatory cytokines and pepsinogen levels in *H. pylori*‐infected patients.

**Method:**

A total of 165 *H. pylori*‐infected subjects without atrophic gastritis were recruited, and their demographic, medical, and lifestyle information were collected. Inflammatory cytokines such as IL‐8, IFN‐*γ*, TNF‐*α*, CRP, IL‐17A, IL‐1*β*, IL‐18, and pepsinogen (PGs I and II) levels were measured. Multiple linear regression was used to analyze the relationship between cytokines and PG levels, whereas segmental regression explored threshold or saturation effects.

**Result:**

IL‐17A and IFN‐*γ* were positively correlated with PG I and LNPG II, with each 1‐pg/mL increase in IL‐17A associated with a 2.595 (95% CI: 0.854, 4.335)‐ng/mL increase in PG I and a 0.012 (95% CI: 0.002, 0.022)‐ng/mL increase in LNPG II; each 1‐pg/mL increase in IFN‐*γ* was associated with a 1.746 (95% CI: 0.702, 2.791)‐ng/mL increase in PG I and a 0.008 (95% CI: 0.002, 0.0191)‐ng/mL increase in LNPG II. No significant correlation was found between inflammatory cytokines and the PG I/PG II ratio (PGR).

**Conclusion:**

In *H. pylori*‐infected patients without atrophic gastritis, IL‐17A and IFN‐*γ* levels showed a linear correlation with PG levels. These cytokines may be closely associated with early gastric mucosal lesions. However, further research is needed to elucidate their specific mechanisms in disease pathogenesis.

## 1. Introduction

*Helicobacter pylori* (HP) is a spiral‐shaped, microaerophilic, gram‐negative bacterium that can cause cellular damage through the production of ammonia and the secretion of substances such as vacuolating toxin A [1]. HP infection can lead to gastric mucosal damage, gastritis, gastric and duodenal ulcers, and even gastric cancer. In 2017, the World Health Organization classified HP as a Group I carcinogen [2]. Epidemiological studies have shown that the global prevalence of HP infection is over 50%, with a rate of 49.6% among adults in China [3]; furthermore, approximately 36.4% of cancer‐related deaths in China can be attributed to digestive tract cancers caused by HP infection [4, 5]. Compared with other regions in China, the HP infection rate is highest in the northwest, reaching 51.8% [6].

The proinflammatory effect of HP is the main mechanism underlying its pathogenic and carcinogenic properties. Lipopolysaccharide on the outer membrane of HP stimulates the human immune system, leading to the production of various proinflammatory cytokines such as interleukin‐1 beta (IL‐1*β*), IL‐6, TNF, interleukin‐8 (IL‐8), interferon‐gamma (IFN‐*γ*), and interleukin‐17A (IL‐17A). These inflammatory factors collectively create the proinflammatory environment characteristic of HP infection [7]. Previous studies have also shown that individuals infected with HP have higher levels of tumor necrosis factor‐alpha (TNF‐*α*), IL‐1*β*, IL‐8, and IFN‐*γ* compared with noninfected individuals, and these levels are highly correlated with the risk of gastric mucosal damage, gastric ulcers, and gastric cancer [8–10]. Only a few studies suggest that in certain HP infection subtypes or early gastritis, the expression of some inflammatory mediators such as IL‐1*β* and IL‐8 may be relatively low, indicating the complexity of the immune response [11].

Pepsinogen (PG), including PGs I and II, is the precursor of gastric pepsin and is commonly used as a biomarker for detecting gastritis (especially atrophic gastritis) and gastric mucosal damage [12]. Changes in PG levels have been observed throughout the development process from HP infection to gastritis and eventually to gastric cancer [13]. In the early stages of gastritis development, the increase in chronic inflammation can lead to elevated levels of PG in individuals infected with HP [14].

Previous in vitro and in vivo studies have found that inflammatory factors play a crucial role in gastric mucosal lesions [15], but there is currently a lack of sufficient population evidence. Therefore, this study is aimed at exploring the relationship between various chronic inflammatory factors and PG in individuals infected with HP but without self‐reported atrophic gastritis. By identifying early inflammatory factors potentially associated with gastric mucosal lesions, this study hopes to provide further population evidence for investigating the pathogenic mechanisms of HP infection and achieving early prevention of gastric mucosal lesions.

## 2. Methods

### 2.1. Participants and Study Design

**Figure 1:**
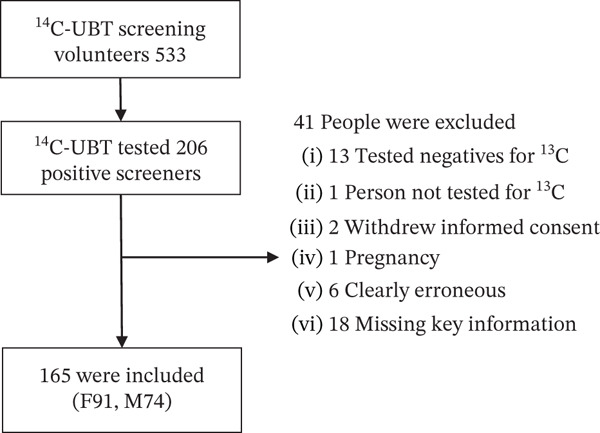
Screening, allocation, and study follow‐up (F, female and M, male).

This study recruited a total of 533 volunteers from the Jingyuan County Traditional Chinese Medicine Hospital and the Third People′s Hospital of Baiyin City, with no gender limitation. The volunteers were required to have no history of previous HP eradication treatment. Among them, 206 individuals tested positive for HP through the ^14^C (^14^C‐UBT) breath test, and they underwent a retest using the ^13^C breath test (^13^C‐UBT) to exclude false‐positive infections. Ultimately, 165 participants were included in the study (Figure 1). The exclusion of atrophic gastritis is based on: The subject self‐reports no history of atrophic gastritis and serum PG testing (PG I ≤ 70 ng/mL or PGR ≤ 6 is considered abnormal) does not indicate atrophic gastritis. All study subjects signed a written informed consent form. This study complied with medical ethics standards and was reviewed and approved by the Ethics Committee of the School of Public Health, Lanzhou University (Ethics Approval Number: IRB19122001).

The inclusion criteria were as follows: (1) age between 20 and 70 years, voluntary participation; (2) positive for HP; (3) body mass index (BMI) between 18 and 30 kg/m^2^; and (4) self‐reported no history of atrophic gastritis.

The exclusion criteria included: (1) refusal to sign the informed consent form; (2) pregnant or lactating women, individuals with allergies; (3) patients with severe cardiovascular, hepatic, renal, hematological disorders, or mental illness; (4) use of medications known to impair gastrointestinal function within the past 3 months; (5) patients using medications (such as nonsteroidal anti‐inflammatory drugs or anticoagulants) that could potentially affect the study; and (6) patients with severe gastrointestinal ulcers.

### 2.2. Data Collection

#### 2.2.1. Questionnaire Survey

We used a self‐designed questionnaire, and the face‐to‐face interviews were conducted by trained investigators. The baseline survey included general information about the subjects, such as age, gender, education level, family annual income, medical history, family history of diseases, smoking status, and drinking habits.

Physical examination includes measurements of blood pressure, heart rate, height, and weight.

#### 2.2.2. Biological Sample Collection

We collected 5 mL of fasting venous blood from the research subjects after an 8‐h fasting period. The blood samples were immediately centrifuged, divided into smaller portions, and stored at −80°C ultralow temperature. Three research subjects were excluded from the analysis due to hemolysis in their blood samples.

### 2.3. HP Infection Detection and Sample Tests

#### 2.3.1. ^13^C‐UBT Test

The subjects were tested for ^13^C‐UBT in the morning on an empty stomach (fasting for 8 h or more), and the test result is expressed as the delta over baseline (DOB) value. The instrument was calibrated with standard gas and quality control gas before each test. A DOB value greater than or equal to 4.0 is considered positive for HP infection.

#### 2.3.2. PG Test

The determination of PGs I and II was performed using a time‐resolved fluorescence immunoassay reagent kit for PG (Pepsinogen Quantitative Determination Kit; Jiangyuan Industrial Technology, Wuxi, Jiangsu, China). The ratio of PGs I–II (PGR) was calculated. The coefficients of variation for PGs I and II were 10.90% and 9.24%, respectively. Abnormal PG levels were defined as PG I ≤70 ng/mL or PGR ≤6.

#### 2.3.3. Inflammatory Factor Test

Enzyme‐linked immunosorbent assay kits (Human ELISA Kit; Elabscience, Wuhan, Hubei, China) were used to detect inflammatory factors IFN‐*γ*, TNF‐*α*, C‐reactive protein (CRP), IL‐17A, IL‐1*β*, IL‐8, and interleukin‐18 (IL‐18). The coefficients of variation for these inflammatory factors and gastrin‐17 were 11.25%, 9.37%, 12.89%, 5.34%, 10.53%, 9.34%, 9.32%, and 14.13%, respectively.

### 2.4. Statistical Analysis

Continuous variables are described by mean ± standard deviation or median (interquartile range), whereas categorical variables are represented by frequency (composition ratio). The normality of all measured data was assessed using normality tests and Q–Q plots. In particular, natural logarithmic transformation was applied to skewed distribution indicators of PG II, CRP, and IL‐1*β*. Multiple linear regression analysis was performed with inflammatory factors as independent variables and gastric PG as the dependent variable. Model 1 was adjusted for basic demographic information such as gender, age, education level, and family annual income. Model 2 further adjusted for smoking, drinking, BMI, blood pressure, family history of gastric cancer, family history of diabetes, family history of hypertension, history of digestive system diseases, recent use of probiotics or cold and pain relievers in the past month, and other factors. Additionally, smoothing curves and segmented regression were used to explore the threshold or saturation effect between inflammatory factors and gastric PG levels, with the same covariates as Model 2.

Sensitivity analysis was conducted by excluding individuals who reported the possibility of atrophic gastritis (gastric PG I ≤ 70 ng/mL or PGR ≤ 6). All analyses were performed using SPSS 26.0 (IBM Corp., Armonk) and Empower (R 4.2.2) software, and a *p* value ≤ 0.05 was considered statistically significant. All statistical analyses were two‐tailed tests with a significance level of *α* = 0.05.

## 3. Results

### 3.1. Baseline Characteristics of the Study Population

A total of 162 research subjects were included in the analysis, of which 73 (45.1%) were male and 89 (54.9%) were female, with an average age of 43.54 ± 12.47 years. The average BMI was 24.11 ± 3.77. Among them, there were 58 subjects with a family history of hypertension and 32 subjects with digestive system diseases, accounting for 35.8% and 32.1%, respectively. Twenty‐two (13.6%) people have taken cold medicine or pain relievers in the past month (Table 1).

**Table 1:** Basic characteristics of subjects.

Variable	Frequency (%)/*m* *e* *a* *n* ± *S* *D*
**Gender**	
Male	73 (45.1)
**Age (years)**	43.38 ± 12.51
**Education**	
Junior high school and below	35 (21.6)
High or polytechnic school	37 (22.8)
Junior college	41 (25.3)
College and above	49 (30.2)
**Annual family income (yuan)**	
~20,000	41 (25.3)
~60,0000	98 (60.5)
~100,000	23 (14.2)
**Currently or ever smoking**	
Yes	33 (20.4)
**Alcohol intake**	
Yes	29 (17.9)
**Family history of stomach cancer**	4 (2.5)
**Family history of diabetes**	15 (9.3)
**Family history of hypertension**	58 (35.8)
**Bacillus licheniformis or probiotics use**	11 (6.8)
**Flu or painkiller medication use**	22 (13.6)
**Suffer from digestive problems**	52 (32.1)
**BMI (kg/m^2^)**	24.11 ± 3.77
**Systolic pressure**	112.49 ± 15.77
**Diastolic blood pressure**	79.40 ± 10.45
**TNF-*α* (pg/mL)**	14.90 ± 4.97
**IFN-*γ* (pg/mL)**	38.40 ± 14.17
**IL-17A (pg/mL)**	19.68 ± 7.77
**IL-8 (pg/mL)**	53.77 ± 23.52
**IL-18 (pg/mL)**	81.02 ± 34.51
**CRP^a^ (ng/mL)**	1109.60 (873.98, 1618.71)
**IL-1*β* ^a^ (pg/mL)**	11.34 (8.81, 14.40)
**PG I (ng/mL)**	177.73 ± 80.41
**PG II^a^ (ng/mL)**	14.86 (11.0, 19.99)
**PGR**	11.50 ± 4.35

^a^This variable is represented by the median (quartiles) after being transformed using the natural logarithm.

3.2 Relationship Between Inflammatory Factors and Gastric PG.

In both Models 1 and 2, the linear regression results showed a positive correlation between IFN‐*γ* and IL‐17A levels and PGs I and II levels. In Model 1, for every one unit increase in IFN‐*γ*, PG I increased by 1.75 units (95% CI, 0.7, 2.79) and LNPG II increased by 0.009 units (95% CI, 0.003, 0.015). For every one unit increase in IL‐17A, PG I increased by 2.89 units (95% CI, 1.16, 4.62) and LNPG II increased by 0.013 units (95% CI, 0.003, 0.023). After further adjustment for covariates in Model 2, for every one unit increase in IFN‐*γ*, PG I increased by 1.746 units (95% CI, 0.702, 2.791) and LNPG II increased by 0.008 units (95% CI, 0.002, 0.014). For every one unit increase in IL‐17A, PG I increased by 2.595 units (95% CI, 0.854, 4.335) and LNPG II increased by 0.012 units (95% CI, 0.002, 0.022). No significant associations were found between other inflammatory factors and PGs I and II, and all inflammatory factors did not show a linear relationship with PGR (Table 2).

**Table 2:** Results of linear regression analysis of inflammatory cytokines and pepsinogen levels.

Cytokines	Model 1						Model 2					
	PG I		LNPG II^a^		PGR		PG I		LNPG II^a^		PGR	
	*β* (*95%* CI)	*p*	*β* (*95%* CI)	*p*	*β* (*95%* CI)	*p*	*β* (*95%* CI)	*p*	*β* (*95%* CI)	*p*	*β* (*95%* CI)	*p*
TNF‐*α* (pg/mL)	1.15 (−1.55, 3.85)	0.402	−0.002 (−0.017, 0.014)	0.807	0.1 (−0.04, 0.24)	0.154	0.98 (−1.66, 3.63)	0.463	−0.002 (−0.017, 0.014)	0.811	0.09 (−0.06, 0.23)	0.226
IFN‐*γ* (pg/mL)	1.75 (0.70, 2.79)	**0.001**	0.009 (0.003, 0.015)	**0.005**	0.01 (−0.05, 0.06)	0.848	1.66 (0.63, 2.69)	**0.002**	0.008 (0.002, 0.014)	**0.008**	0.01 (−0.05, 0.06)	0.874
IL‐17A (pg/mL)	2.89 (1.16, 4.62)	**0.001**	0.013 (0.003, 0.023)	**0.013**	0.04 (−0.05, 0.14)	0.347	2.6 (0.85, 4.34)	**0.004**	0.012 (0.002, 0.022)	**0.025**	0.04 (−0.06, 0.13)	0.453
IL‐8 (pg/mL)	0.08 (−0.48, 0.63)	0.79	0.00 (−0.003, 0.003)	0.865	0.01 (−0.02, 0.04)	0.512	0.06 (−0.48, 0.61)	0.822	0 (−0.003, 0.004)	0.821	0.01 (−0.02, 0.04)	0.618
IL‐18 (pg/mL)	0.23 (−0.14, 0.6)	0.225	0.002 (−0.001, 0.004)	0.147	0.00 (−0.02, 0.02)	0.733	0.2 (−0.17, 0.58)	0.288	0.002 (−0.001, 0.004)	0.138	0.00 (−0.02, 0.02)	0.941
LNCRP^a^ (ng/mL)	−4.46 (−31.59, 22.67)	0.746	−0.067 (−0.223, 0.089)	0.397	0.83 (−0.58, 2.23)	0.247	−3.16 (−31.37, 25.04)	0.825	−0.049 (−0.214, 0.116)	0.561	0.76 (−0.76, 2.29)	0.325
LNIL‐1*β* ^a^ (pg/mL)	25.37 (−8.12, 58.85)	0.137	0.007 (−0.187, 0.202)	0.941	1.13 (−0.61, 2.87)	0.202	17.86 (−14.96, 50.68)	0.284	−0.022 (−0.215, 0.17)	0.819	1.01 (−0.77, 2.79)	0.263

*Note:* Model 1: adjusted for gender, age, family annual income, and education level. Model 2: further adjusted for smoking status, drinking status, systolic blood pressure, diastolic blood pressure, family history of gastric cancer, family history of hypertension, family history of diabetes, digestive system diseases, recent use of cold medicine, use of probiotics, and BMI.

^a^The data are transformed using a natural logarithm (LN).

Through fitting smooth curves (Figures S1, S2, and S3) and segmented regression analyses, no nonlinear threshold or saturation effects were observed between all inflammatory factors and levels of gastric PG, and all inflammatory factors did not show a linear relationship with PGR. When fitting the data with a straight line, for every 1 pg/mL increase in IFN‐*γ*, PG I and LNPG II increased by 1.67 (95% CI, 0.64, 2.71) ng/mL and 0.01 (95% CI, 0.00, 0.01) ng/mL, respectively (*p* = 0.002, 0.020), and no threshold effect was found between IFN‐*γ* and PG (*p* = 0.159, 0.112). Similarly, when fitting the data with a straight line, for every 1 pg/mL increase in IL‐17A, PG I and LNPG II increased by 2.79 (95% CI, 1.07, 4.50) ng/mL and 0.01 (95% CI, 0.00, 0.02) ng/mL, respectively (*p* = 0.002, 0.027), and there was no statistically significant difference in the threshold effect analysis (*p* = 0.372, 0.088) (Table 3).

**Table 3:** Analysis of the threshold effect between inflammatory cytokines pepsinogen levels.

	Model 1 one line slope	Model 2 turning point (K)		*p*
PG I	***β* (*95%* CI)**	***β* (*95%* CI)**		
TNF‐*α* (pg/mL)	One line slope	< 21.82 Slope 1	> 21.82 Slope 2	0.170
	0.54 (−2.11, 3.18)	1.84 (−1.47, 5.14)	−5.07 (−14.07, 3.92)	
IFN‐*γ* (pg/mL)	One line slope	< 49.13 Slope 1	>49.13 Slope 2	0.159
	**1.67 (0.64, 2.71)** ^∗∗^	**2.60 (0.87, 4.33)** ^∗∗^	0.42 (−1.72, 2.56)	
IL‐17A (pg/mL)	One line slope	< 10.30 Slope 1	>10.30 Slope 2	0.372
	**2.79 (1.07, 4.50)** ^∗∗^	12.42 (−10.38, 35.22)	**2.62 (0.86, 4.38)** ^∗∗^	
IL‐8 (pg/mL)	One line slope	< 95.30 Slope 1	> 95.30 Slope 2	0.156
	0.0 (−0.5, 0.6)	−0.3 (−1.0, 0.4)	1.5 (−0.8, 3.7)	
IL‐18 (pg/mL)	One line slope	<40.60 Slope 1	> 40.60 Slope 2	0.075
	0.2 (−0.2, 0.6)	−3.1 (−7.0, 0.8)	0.3 (−0.1, 0.7)	
LNCRP^a^ (ng/mL)	One line slope	< 6.85 Slope 1	> 6.85 Slope 2	0.102
	−4.89 (−32.62, 22.84)	66.26 (−29.18, 161.70)	−20.97 (−55.43, 13.50)	
LNIL‐1*β* ^a^ (pg/mL)	One line slope	< 2.26 Slope 1	> 2.26 Slope 2	0.148
	13.25 (−20.14, 46.61)	‐38.26 (−120.92, 44.40)	39.97 (−11.48, 91.42)	
PG II				
TNF‐*α* (pg/mL)	One line slope	< 21.45 Slope 1	> 21.45 Slope 2	0.066
	0.00 (−0.02, 0.01)	0.01 (−0.01, 0.03)	−0.05 (−0.10, 0.00)	
IFN‐*γ* (pg/mL)	One line slope	< 51.76 Slope 1	> 51.76 Slope 2	0.112
	**0.01 (0.00, 0.01)** ^∗^	**0.01 (0.00, 0.02)** ^∗∗^	−0.00 (−0.02, 0.01)	
IL‐17A (pg/mL)	One line slope	< 18.10 Slope 1	> 18.10 Slope 2	0.088
	**0.01 (0.00, 0.02)** ^∗^	**0.04 (0.00, 0.07)** ^∗^	0.00 (−0.01, 0.02)	
IL‐8 (pg/mL)	One line slope	< 48.16 Slope 1	> 48.16 Slope 2	0.335
	0.00 (−0.00, 0.00)	−0.00 (−0.01, 0.01)	0.00 (−0.00, 0.01)	
IL‐18 (pg/mL)	One line slope	< 42.00 Slope 1	> 42.00 Slope 2	0.082
	0.00 (−0.00, 0.00)	−0.02 (−0.04, 0.01)	0.00 (−0.00, 0.00)	
LNCRP^a^ (ng/mL)	One line slope	< 6.51 Slope 1	> 6.51 Slope 2	0.102
	−0.06 (−0.23, 0.10)	0.74 (−0.58, 2.07)	−0.09 (−0.27, 0.08)	
LNIL‐1*β* ^a^ (pg/mL)	One line slope	< 2.97 Slope 1	> 2.97 Slope 2	0.308
	−0.05 (−0.25, 0.14)	−0.00 (−0.23, 0.22)	−0.50 (−1.45, 0.44)	
Continued Table 3 Analysis of the threshold effect between inflammatory cytokines pepsinogen levels				
	**Model 1 One line slope**	**Model 2 Turning point (K)**		***P***
PGR	***β* (*95%* CI)**	***β* (*95%* CI)**		
TNF‐*α* (pg/mL)	One line slope	< 8.67 Slope 1	> 8.67 Slope 2	0.070
	0.08 (−0.06, 0.22)	1.46 (−0.14, 3.06)	0.04 (−0.11, 0.19)	
IFN‐*γ* (pg/mL)	One line slope	< 21.08 Slope 1	> 21.08 Slope 2	0.383
	0.01 (−0.05, 0.06)	0.30 (−0.42, 1.02)	0.00 (−0.06, 0.06)	
IL‐17A (pg/mL)	One line slope	< 18.06 Slope 1	> 18.06 Slope 2	0.066
	0.04 (−0.05, 0.14)	−0.19 (−0.48, 0.10)	0.12 (−0.01, 0.25)	
IL‐8 (pg/mL)	One line slope	< 69.48 Slope 1	> 69.48 Slope 2	0.239
	0.01 (−0.02, 0.04)	−0.02 (−0.07, 0.03)	0.04 (−0.03, 0.10)	
IL‐18 (pg/mL)	One line slope	< 81.83 Slope 1	> 81.83 Slope 2	0.423
	0.00 (−0.02, 0.02)	−0.02 (−0.04, 0.01)	0.01 (−0.02, 0.04)	
LNCRP^a^ (ng/mL)	One line slope	< 6.85 Slope 1	> 6.85 Slope 2	0.175
	0.76 (−0.75, 2.28)	3.99 (−1.24, 9.22)	0.03 (−1.85, 1.92)	
LNIL‐1*β* ^a^ (pg/mL)	One line slope	< 2.97 Slope 1	> 2.97 Slope 2	0.101
	1.00 (−0.80, 2.79)	0.26 (−1.76, 2.28)	7.60 (−1.03, 16.24)	

*Note:* *p* there is a significant difference between Models 1 and 2; the models adjust for gender, age, household income, education level, smoking, drinking, systolic blood pressure, diastolic blood pressure, family history of stomach cancer, family history of hypertension, family history of diabetes, digestive system diseases, use of cold medicine, use of probiotics, and BMI.

^a^The data are transformed using natural logarithm.

^∗^ < 0.05.

^∗∗^ < 0.01.

^∗∗∗^ < 0.001.

Furthermore, sensitivity analysis by excluding study subjects who may have atrophic gastritis lesions (PG I ≤ 70 or PGR ≤ 6) also revealed a positive correlation between IFN‐*γ* and IL‐17A levels and PGs I and II levels, whereas IL‐17A showed a marginal statistical linear relationship with PG II in Model 1. No other inflammatory factors were found to be related to PG in the sensitivity analysis (Table S1).

## 4. Discussion

In this study, we found a positive correlation between IL‐17A and IFN‐*γ* with gastric PGs I and II. As IL‐17A and IFN‐*γ* levels increased, the levels of PGs I and II also gradually rose. However, other inflammatory markers did not show any correlation with PGs I and II.

HP infection is the most common cause of various gastrointestinal diseases such as chronic gastritis, atrophic gastritis, and gastric cancer. It interacts with host cells in the gastric mucosa, activating multiple immune signaling pathways and producing various proinflammatory cytokines [9, 10], leading to changes in gastric mucosal state and stimulation of the release of PG [16, 17]. Previous studies have shown that serum PG concentration can effectively evaluate gastritis, gastric atrophy, and gastric mucosal integrity [12]. After HP infection, the level of PG increases with the severity of gastric body inflammation and decreases after gastric atrophy [15]. However, there have been few reports on the relationship between inflammatory factors before the occurrence of atrophic gastritis and PG, and it is still unclear which inflammatory factors are reflected by PG. This study suggests that IL‐17A and IFN‐*γ* may be the main effector factors of gastric inflammation reflected by PG after HP infection. Adamsson et al. also drew the same conclusion in a small‐sample nonrandomized cross‐sectional study with 44 subjects, indicating that the response of T cells to HP infection may mainly involve the infiltration of Th1 and T helper 17 (Th17) CD4+ T cells, resulting in the production of IFN‐*γ* and IL‐17A [18, 19].

IL‐17A is an inflammatory cytokine secreted by CD4+ Th17 cells and other immune cells. After infection with HP, the levels of IL‐17A and IFN‐*γ* in the gastric mucosa are upregulated. During the early stage of infection, IL‐17A production induces the activation of neutrophils to eliminate bacteria, but in the later stage, it can lead to chronic inflammation, thereby favoring pathogen infection [20, 21]. Chronic inflammation can cause atrophy and metaplasia of epithelial cells, increasing the risk of gastric cancer [17]. Previous studies have shown that IL‐17A is expressed in both gastric cancer tissue and normal gastric mucosa tissue, but its expression in gastric cancer tissue is significantly higher than that in normal gastric mucosa tissue [22]. However, the mechanism of IL‐17A in the occurrence of gastric atrophy before gastric cancer is not well‐studied. The development of gastric cancer involves a complex, multistep process with multiple mechanisms. Chronic atrophic gastritis is one of the significant precancerous conditions, but not all cases of atrophic gastritis progress to gastric cancer. Zong et al. found through in vitro studies that HP induces MHCII+ monocytes to secrete IL‐23, which helps polarize CD4+ cells (Th17 cells) that produce IL‐17A, thereby promoting the occurrence of HP‐related gastritis [23]. Mouse experiments conducted by Bockerstett et al. further elucidated that IL‐17A is a cytokine that promotes apoptosis of wall cells during atrophic gastritis (precancerous lesion of gastric cancer). In the chronic inflammation process caused by excessive production of IL‐17A, apoptosis promotes the development of atrophy and metaplasia [24]. A small‐sample human controlled trial conducted by Adamsson also found that the elevation of IL‐17A, apart from promoting inflammation, may also lead to the occurrence of malignant gastric ulcers [18]. Our study found that the elevation of IL‐17A in individuals infected with HP is closely correlated with the elevation of PGs I and II, indicating that IL‐17A may play an important role in the development of atrophic gastritis or gastric ulcers caused by HP infection. When individuals infected with HP have persistently elevated levels of gastric PG, inhibiting IL‐17A levels may potentially prevent or delay the occurrence of gastric ulcers and atrophy, and it can be used as a potential means to treat gastric cancer [25]. However, it is worth noting that a study by Cremniter suggests that although IL‐17A is expressed at higher levels in individuals infected with HP, it does not have a specific relationship with the occurrence of the disease [26], Therefore, the degree of IL‐17A′s involvement in gastric diseases still needs to be further confirmed by higher‐level evidence.

IFN‐*γ* is the main effector factor secreted by Th1 cells, which can induce inflammatory damage to epithelial tissues and increase the risk of malignant transformation of epithelial cells [27]. It plays an important role in the proliferation, migration, and angiogenesis of gastric cancer [28]. Although the role of HP infection in chronic inflammation is not clear yet, recent studies combining human and animal models have suggested that catecholamines directly promote T cell proliferation and the production of IFN‐*γ* in gastric epithelial cells infected with HP. Meanwhile, it also indirectly acts on T cells by stimulating macrophages to produce IL‐12. This direct and indirect effect leads to the development of chronic inflammation in individuals infected with HP. In addition, this study found that knocking out the IFN gene in HP‐infected mice can inhibit the development of inflammation [28]. Osaki et al. also found in animal models that IFN‐*γ* is a critical initiating factor in the development of gastritis to gastric atrophy and metaplasia [29]. Bagheri et al.′s cross‐sectional study also found a positive correlation between the expression level of IFN‐*γ* and the density of HP and the degree of chronic inflammation. However, this study found that the content of CD4+ T cells was low in patients with gastritis and gradually increased in patients with gastric ulcers. Moreover, IL‐17A and IFN‐*γ* were found to be higher in HP‐infected individuals with peptic ulcers than those with gastritis [30], This may be due to the fact that IFN‐*γ* induces functional changes in gastric epithelial cells, thereby increasing gastric acid secretion [31]. This suggests that when PGs I and II increase synchronously while PGR remains unchanged, the risk of developing peptic ulcers may be higher in individuals infected with HP. However, different from our study, Kang et al.only found in human cell experiments that IFN‐*γ* can induce the secretion of PG II but not PG I [32], so further research is still needed to clarify the relationship between IFN‐*γ* and PG I.

In addition, in vitro studies by Serrano et al. from Spain suggest a correlation between the secretion of IL‐1*β* and gastric zymogen release [33]. A cohort study conducted by Krishnan et al. from Australia showed that children with congenital esophageal atresia had high levels of inflammatory factors such as IL‐1*β* and IL‐8, accompanied by elevated levels of gastric PG A3 [34]. However, this study did not find any association between these inflammatory factors and gastric PG levels, possibly due to the different disease stages of the study subjects. Most of the subjects in Serrano et al.′s study (*n* = 60) had other gastrointestinal diseases such as duodenitis and gastric ulcers, and they were predominantly male. On the other hand, Krishnan et al.′s study subjects mainly had gastroesophageal reflux, and the strong inflammatory response may have contributed to the changes in gastric PG. In our study, the majority of the subjects did not have severe gastric mucosal diseases. Furthermore, the differences in gut microbiota among different races or geographical populations may also explain why we did not observe these associations. For example, a small‐scale study in Korea suggested that changes in gut microbiota due to the consumption of fermented kimchi contributed to the improvement of HP‐related chronic atrophic gastritis and significantly increased PGR release [35].

The main advantages of this study are as follows: firstly, the study conducted two HP detection breath tests, which reduced the false positive rate of HP infection in the study population. Secondly, the study subjects were HP‐infected individuals without atrophic gastritis in high‐risk areas for gastric cancer, which has exploratory significance in preventing atrophic gastritis and gastric cancer in HP‐infected individuals. Additionally, we conducted more tests on inflammatory cytokines and related biomarkers compared to published studies, which to some extent reduced subjectivity. The limitations of this study include a small sample size, which limited our ability to explore the association between low‐sensitive inflammatory factors and gastric protease levels, and the ability to explore sensitive populations through stratified analysis. Furthermore, this study with sample size is determined based on clinical recruitment feasibility rather than prior estimation. Future research is advised to conduct prospective validation and complete prior sample size calculations. The study subjects were all HP‐infected individuals without apparent health issues, so the results may not be applicable to individuals with severe gastric mucosal lesions. Cross‐sectional studies cannot determine causal relationships. All study subjects were recruited from hospitals, which inevitably introduced selection bias. Although gastric protease can reflect gastric mucosal function and status to some extent, it cannot accurately reflect the degree of gastric mucosal lesions like histopathological examination. The implementation of histopathological examination was limited due to the majority of study participants being apparently healthy individuals, which limits the application of the study results.

## 5. Conclusion

This study found a linear correlation between IL‐17A and IFN‐*γ* levels and PG in HP‐infected individuals without atrophic gastritis. IL‐17A and IFN‐*γ* may be closely associated with early mucosal lesions, providing evidence for further in‐depth research on the early pathogenic mechanisms of gastric mucosal lesions caused by HP infection and early prevention of gastric mucosal lesions.

## Author Contributions

Li Minghong proposed the main research objectives. Li Minghong and Guo Kai were responsible for conducting experiments and implementing the research. Yang Dongmei and Tian Chiyu were responsible for processing, performing statistical analysis, and writing the paper. Li Minghong, Guo Kai, Pan Wei, and Lv Runhua collected and organized the data. Jing Lipeng provided funding support and was responsible for quality control and review of the article. Yang Dongmei was the overall responsible for the article and provided supervision and management. Li Minghong and Tian Chiyu contributed equally.

## Funding

No funding was received for this manuscript.

## Ethics Statement

This study was conducted in accordance with the Declaration of Helsinki and approved by the Ethics Committee of the School of Public Health of Lanzhou University (Approval No. IRB19122001).

## Consent

Informed consent was obtained from all individual participants included in the study. Details that might disclose the identity of the subjects under study have been omitted.

## Conflicts of Interest

The authors declare no conflicts of interest.

## Supporting information


**Supporting Information** Additional supporting information can be found online in the Supporting Information section. Table S1: Sensitivity analysis of the associations between specific inflammatory cytokines and pepsinogen levels using multiple linear regression. The table presents regression coefficients (*β*), 95% confidence intervals (95% CI), and *p* values for the associations of TNF‐*α*, IL‐17A, IL‐8, IL‐18, IFN‐*γ*, high‐sensitivity C‐reactive protein (CRP), IL‐1*β* with Pepsinogen I (PG I), natural log‐transformed Pepsinogen II (LN PG II), and the pepsinogen ratio (PGR). Analyses were conducted under two adjustment models: Model 1 was adjusted for basic demographic factors (gender, age, family annual income, and education level), and Model 2 was further adjusted for lifestyle and clinical factors (smoking status, drinking status, systolic and diastolic blood pressure, family history of gastric cancer, hypertension, and diabetes, personal history of digestive system diseases, recent use of cold medicine, use of probiotics, and BMI). Figure S1: Smooth curve fitting plots for the relationships between individual inflammatory cytokines (TNF‐*α*, IL‐17A, IL‐8, IL‐18, IFN‐*γ*, CRP, IL‐1*β*) and Pepsinogen I (PG I) levels. The solid lines represent the fitted curves from the nonparametric regression, which are used to visually assess the shape and potential nonlinearity of each association after adjusting for covariates. Figure S2: Smooth curve fitting plots for the relationships between individual inflammatory cytokines (TNF‐*α*, IL‐17A, IL‐8, IL‐18, IFN‐*γ*, CRP, and IL‐1*β*) and Pepsinogen II (PG II) levels. The solid lines represent the fitted curves from the nonparametric regression, which are used to visually assess the shape and potential nonlinearity of each association after adjusting for covariates. Figure S3: Smooth curve fitting plots for the relationships between individual inflammatory cytokines (TNF‐*α*, IL‐17A, IL‐8, IL‐18, IFN‐*γ*, CRP, and IL‐1*β*) and the pepsinogen ratio (PGR). The solid lines represent the fitted curves from the nonparametric regression, which are used to visually assess the shape and potential nonlinearity of each association after adjusting for covariates.

## Data Availability

The data generated in this study are available upon request from the corresponding author.
