# Supplementary material for: Inflammatory Cytokines as Potential Inducements of Early Gastric Mucosal Lesions in Helicobacter pylori‐Infected Patients
Source: Gastroenterol Res Pract. 2026 May 18;2026:9063312. doi: 10.1155/grp/9063312 (PMC13181917; doi:10.1155/grp/9063312)
Supplement: Supplementary file 1 — Supporting Information Additional supporting information can be found online in the Supporting Information section. Table S1: Sensitivity analysis of the associations between specific inflammatory cytokines and pepsinogen levels using multiple linear regression. The table presents regression coefficients (β), 95% confidence intervals (95% CI), and p values for the associations of TNF‐α, IL‐17A, IL‐8, IL‐18, IFN‐γ, high‐sensitivity C‐reactive protein (CRP), IL‐1β with Pepsinogen I (PG I), natural log‐transformed Pepsinogen II (LN PG II), and the pepsinogen ratio (PGR). Analyses were conducted under two adjustment models: Model 1 was adjusted for basic demographic factors (gender, age, family annual income, and education level), and Model 2 was further adjusted for lifestyle and clinical factors (smoking status, drinking status, systolic and diastolic blood pressure, family history of gastric cancer, hypertension, and diabetes, personal history of digestive system diseases, recent use of cold medicine, use of probiotics, and BMI). Figure S1: Smooth curve fitting plots for the relationships between individual inflammatory cytokines (TNF‐α, IL‐17A, IL‐8, IL‐18, IFN‐γ, CRP, IL‐1β) and Pepsinogen I (PG I) levels. The solid lines represent the fitted curves from the nonparametric regression, which are used to visually assess the shape and potential nonlinearity of each association after adjusting for covariates. Figure S2: Smooth curve fitting plots for the relationships between individual inflammatory cytokines (TNF‐α, IL‐17A, IL‐8, IL‐18, IFN‐γ, CRP, and IL‐1β) and Pepsinogen II (PG II) levels. The solid lines represent the fitted curves from the nonparametric regression, which are used to visually assess the shape and potential nonlinearity of each association after adjusting for covariates. Figure S3: Smooth curve fitting plots for the relationships between individual inflammatory cytokines (TNF‐α, IL‐17A, IL‐8, IL‐18, IFN‐γ, CRP, and IL‐1β) and the pepsinogen ratio (P [file GRP-2026-9063312-s001.docx]

**Correlation Between Chronic Inflammatory Cytokines and** **Pepsinogen Levels in Patients with Helicobacter Pylori Infection**

Minghong LI ^a#^, Chiyu TIAN ^b#^, Kai GUO ^c^, Wei PAN ^d^, Runhua LV^d^, Lipeng JING^b^, Dongmei Yang ^e*^

*^a^* Department of clinical laboratory, Jing Yuan County Hospital of Traditional Chinese Medicine, Baiyin, China

*^b^* Institute of Epidemiology and Statistics, School of Public Health, Lanzhou University, Lanzhou, China

*^c^* Department of Cardiology and Cardiovascular Research Institute, Renmin Hospital of Wuhan University, Wuhan, Hubei, China

*^d^* Department of Public Health, Jing Yuan County Hospital of Traditional Chinese Medicine, Baiyin, China

*^e^* Jing Yuan County Hospital of Traditional Chinese Medicine, Baiyin, China

*Corresponding author: Dongmei Yang, Email: 1742398585@qq.com.

#These authors contributed equally

Table S1 Results of sensitivity linear regression analysis of inflammatory cytokines and pepsinogen levels

| Cytokines |  | **Model 1** |  |  |  | **Model 2** |  |
| --- | --- | --- | --- | --- | --- | --- | --- |
|  | *β* | *95%CI* | *P* |  | *β* | *95%CI* | *P* |
| **PG I** |  |  |  |  |  |  |  |
| **TNF-α** | 0.48 | -2.15,3.12 | 0.717 |  | 0.26 | -2.27,2.79 | 0.837 |
| **IL-17A** | 2.02 | 0.29,3.75 | **0.022** |  | 1.63 | -0.08,3.34 | **0.061** |
| **IL-8** | 0.12 | -0.42,0.67 | 0.658 |  | 0.07 | -0.46,0.6 | 0.797 |
| **IL-18** | 0.30 | -0.1,0.68 | 0.137 |  | 0.28 | -0.1,0.67 | 0.151 |
| **IFN-γ** | 1.27 | 0.24,2.3 | **0.016** |  | 1.17 | 0.16,2.17 | **0.023** |
| **LNCRP^a^** | -7.55 | -33.92,18.82 | 0.572 |  | -7.90 | -35.3,19.5 | 0.569 |
| **LNIL-1β^a^** | 17.32 | -15.04,49.67 | 0.292 |  | 9.71 | -21.41,40.83 | 0.538 |
| **LNPG Ⅱ** |  | , |  |  |  | , |  |
| **TNF-α** | 0.02 | -0.24,0.28 | 0.874 |  | 0.02 | -0.23,0.28 | 0.869 |
| **IL-17A** | 0.17 | 0.00,0.35 | **0.052** |  | 0.15 | -0.03,0.32 | 0.093 |
| **IL-8** | 0.01 | -0.05,0.06 | 0.822 |  | 0.01 | -0.05,0.06 | 0.863 |
| **IL-18** | 0.02 | -0.02,0.06 | 0.264 |  | 0.02 | -0.02,0.06 | 0.225 |
| **IFN-γ** | 0.15 | 0.05,0.25 | **0.004** |  | 0.15 | 0.05,0.25 | **0.005** |
| **LNCRP^a^** | -1.35 | -3.97,1.27 | 0.31 |  | -1.28 | -4.03,1.48 | 0.360 |
| **LNIL-1β^a^** | 1.64 | -1.59,4.86 | 0.318 |  | 1.09 | -2.05,4.22 | 0.494 |
| **PGR** |  | , |  |  |  | , |  |
| **TNF-α** | 0.07 | -0.07,0.2 | 0.307 |  | 0.05 | -0.09,0.19 | 0.460 |
| **IL-17A** | 0.02 | -0.07,0.11 | 0.699 |  | 0.00 | -0.09,0.1 | 0.929 |
| **IL-8** | 0.01 | -0.02,0.04 | 0.487 |  | 0.01 | -0.02,0.03 | 0.671 |
| **IL-18** | 0.01 | -0.01,0.03 | 0.579 |  | 0.00 | -0.02,0.02 | 0.980 |
| **IFN-γ** | -0.01 | -0.07,0.04 | 0.594 |  | -0.02 | -0.08,0.03 | 0.436 |
| **LNCRP^a^** | 0.51 | -0.84,1.85 | 0.457 |  | 0.31 | -1.17,1.79 | 0.679 |
| **LNIL-1β^a^** | 0.51 | -1.14,2.17 | 0.54 |  | 0.26 | -2.27,2.79 | 0.760 |

a, the data is transformed using a natural logarithm (LN); Model 1: adjusted for gender, age, family annual income, and education level; Model 2: further adjusted for smoking status, drinking status, systolic blood pressure, diastolic blood pressure, family history of gastric cancer, family history of hypertension, family history of diabetes, digestive system diseases, recent use of cold medicine, use of probiotics, BMI.


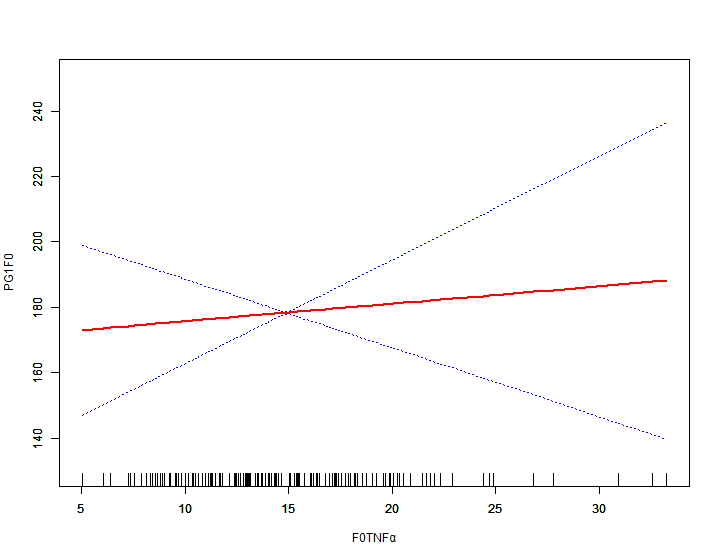

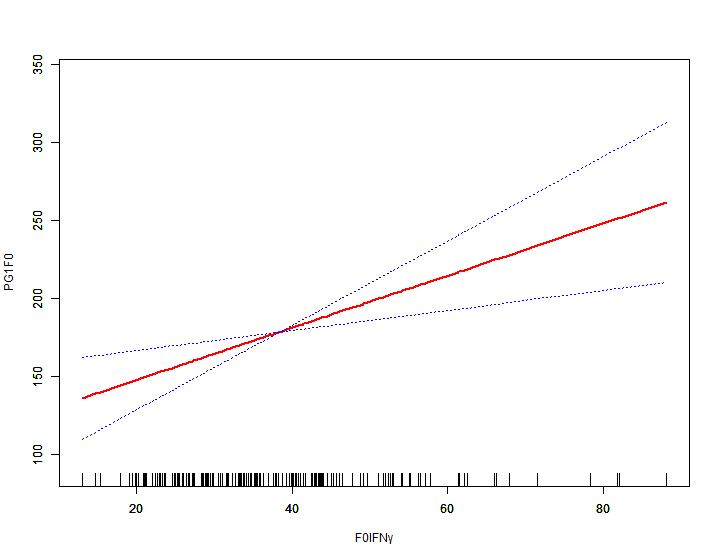

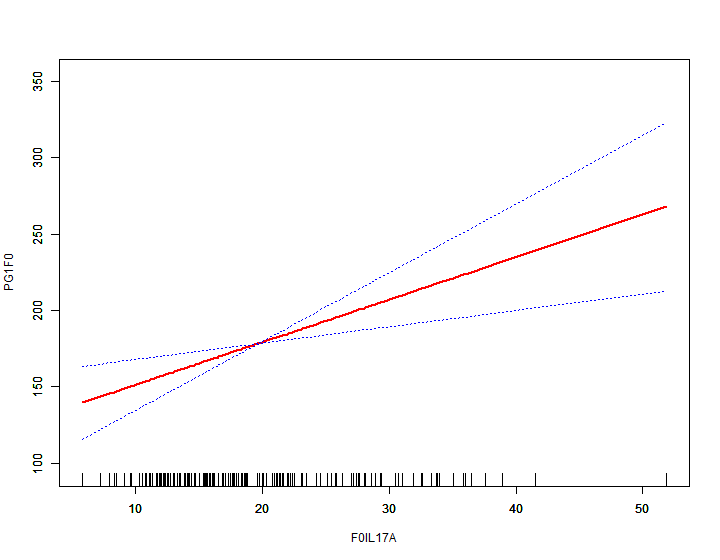

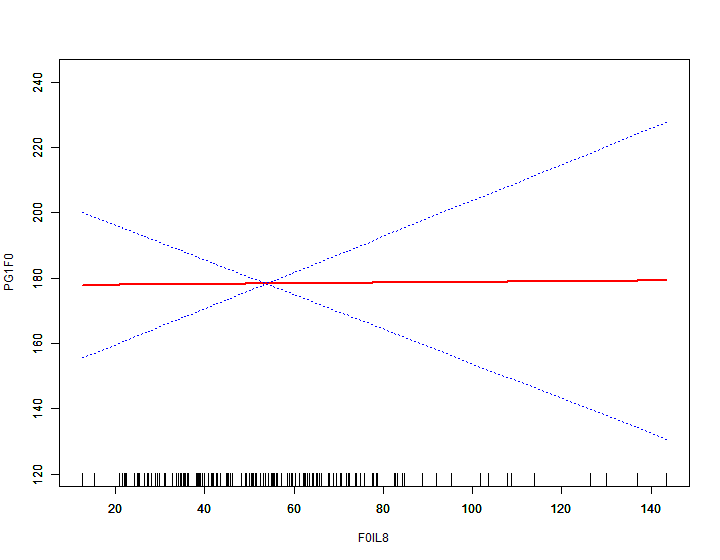

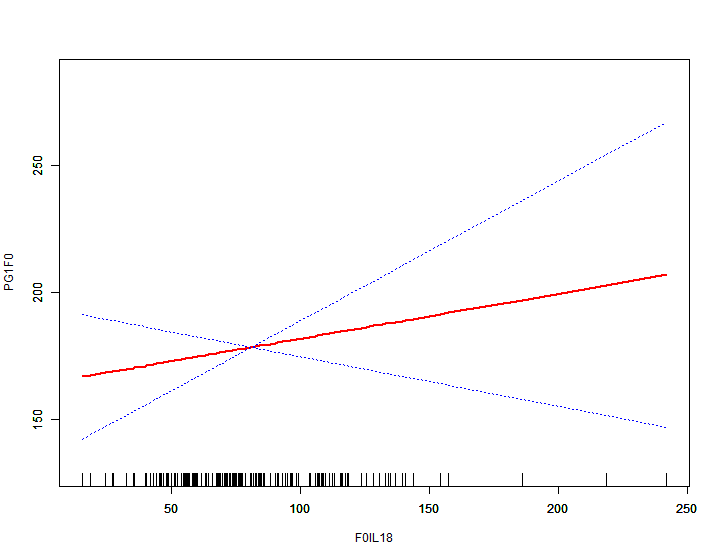

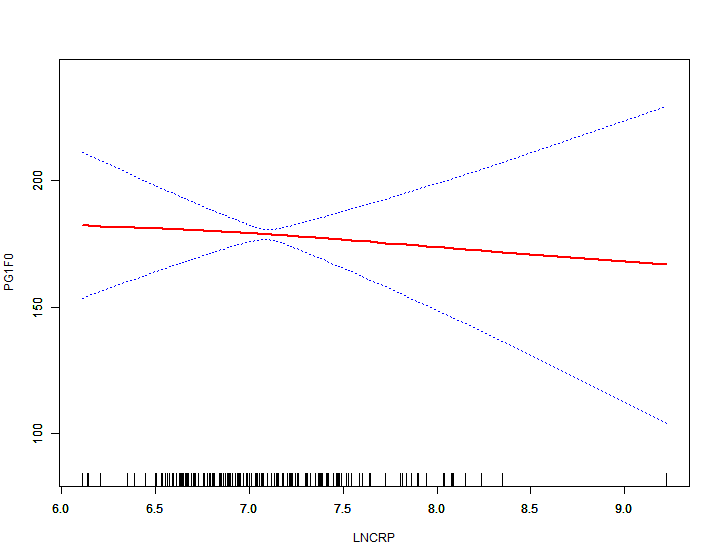

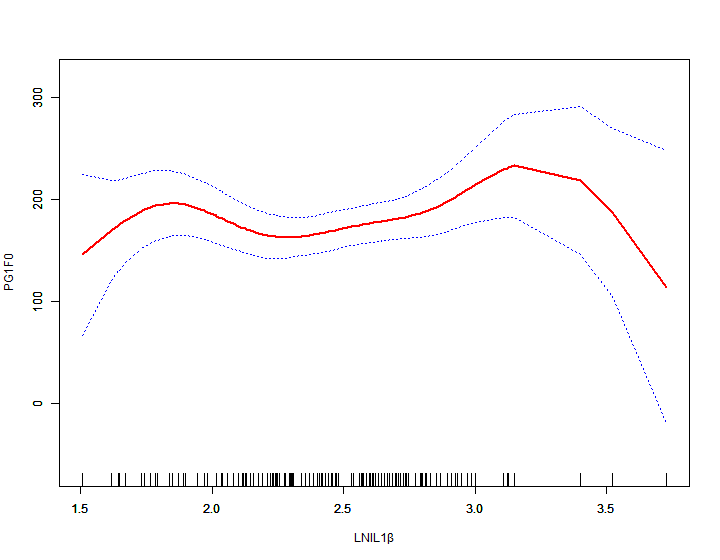


Figure S1 Inflammatory factors and PG Ⅰ levels smooth curve fitting

Figure S2 Inflammatory factors and PG Ⅱ levels smooth curve fitting


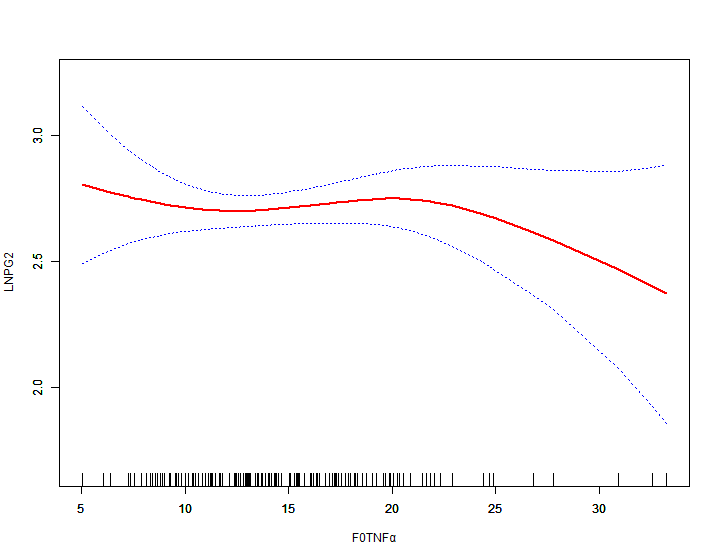

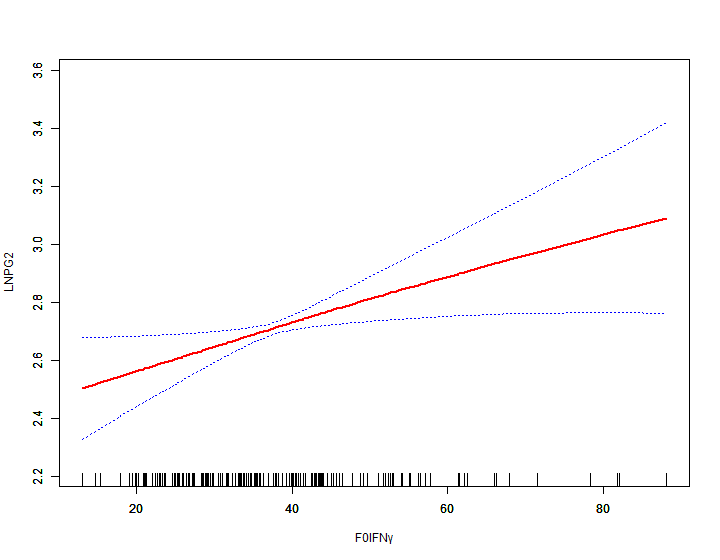

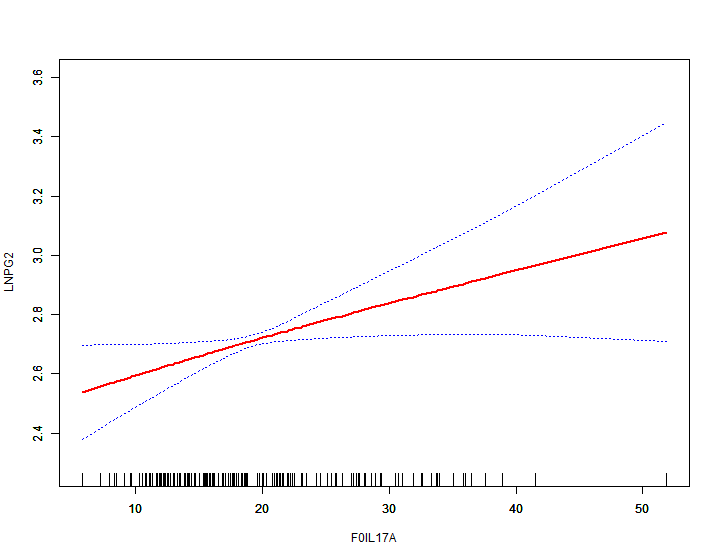

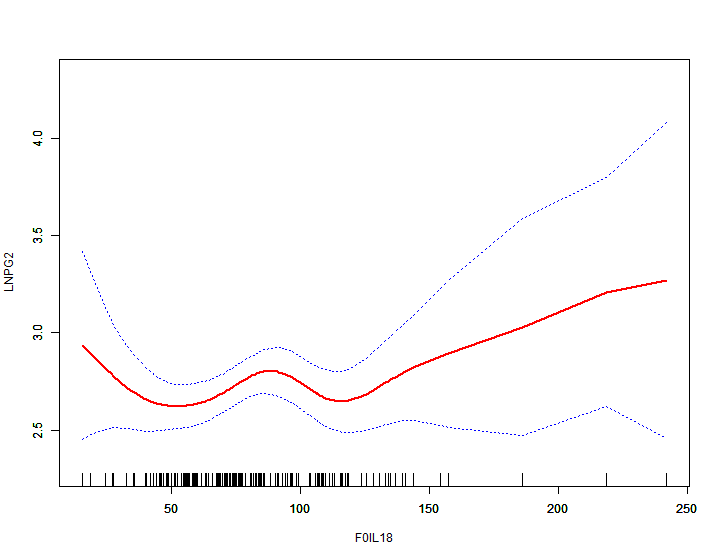

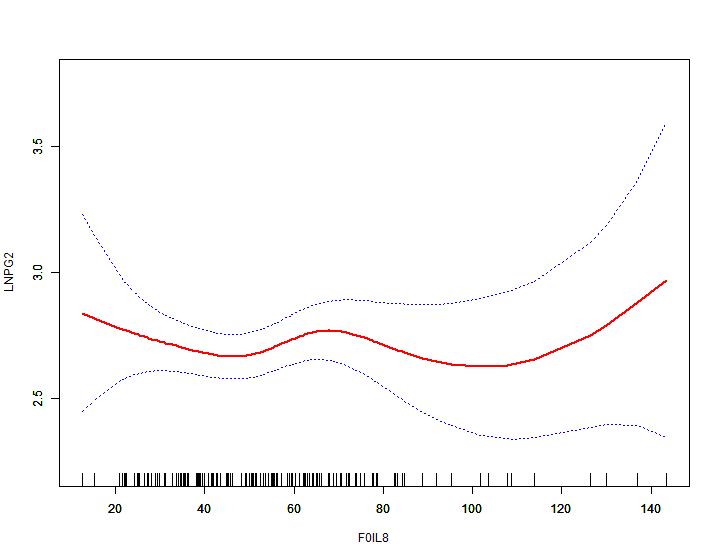

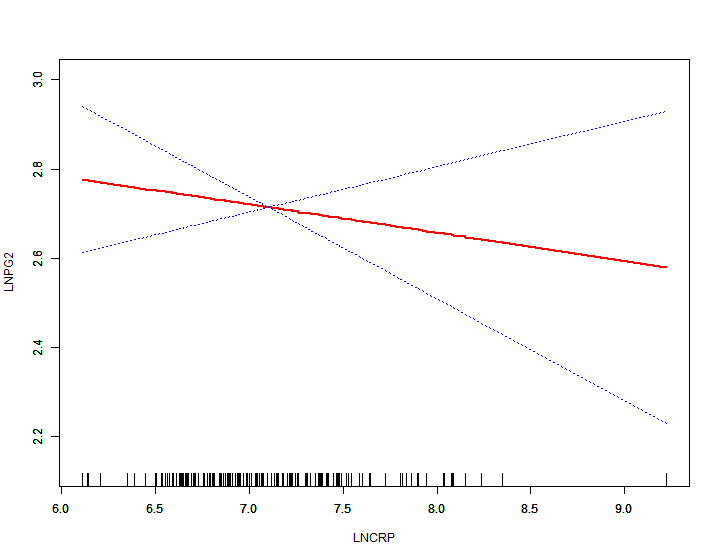

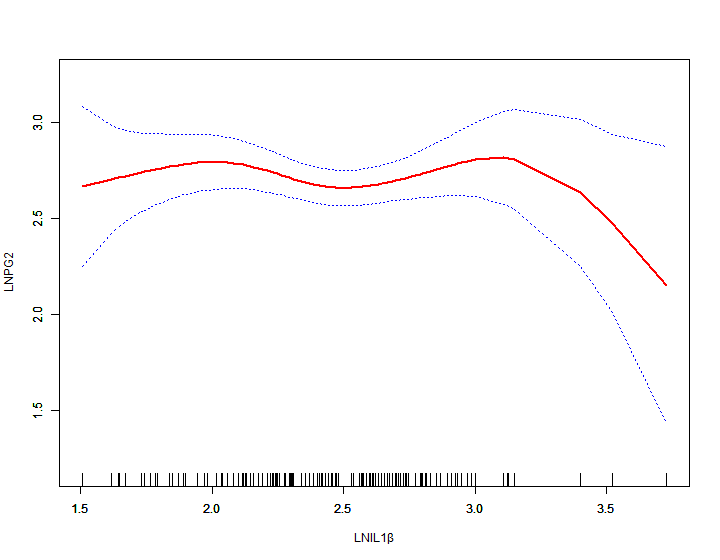

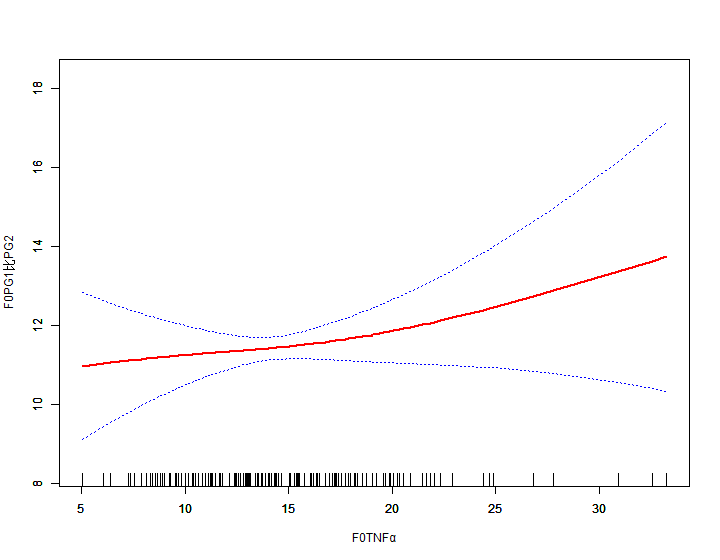

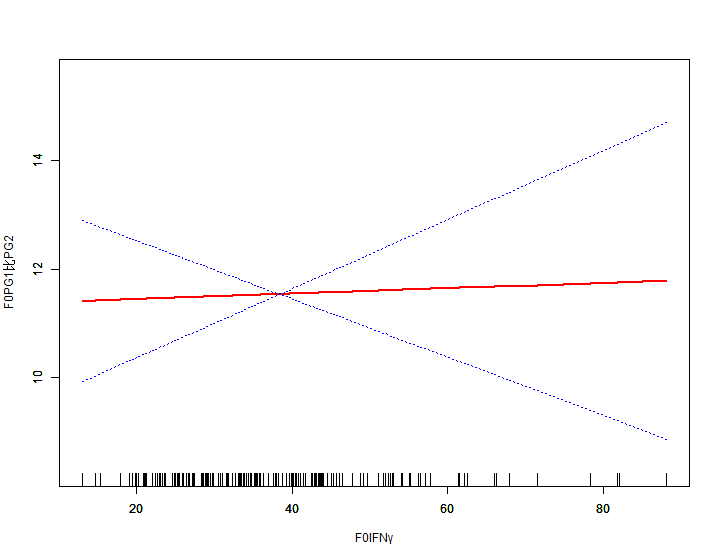

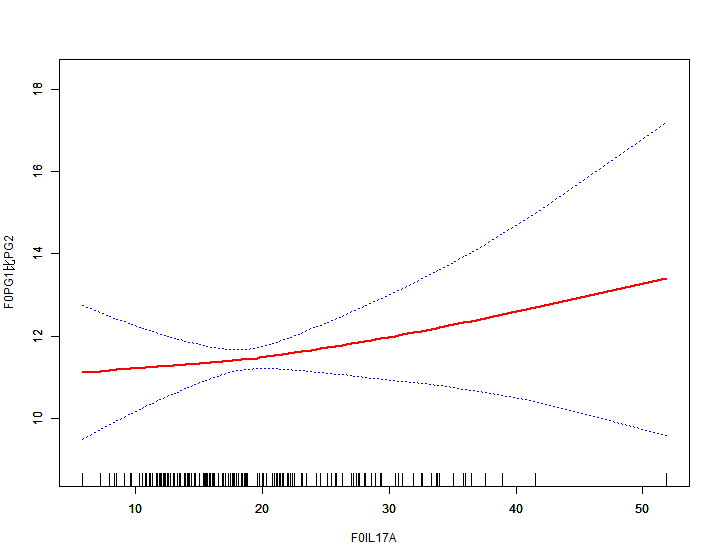

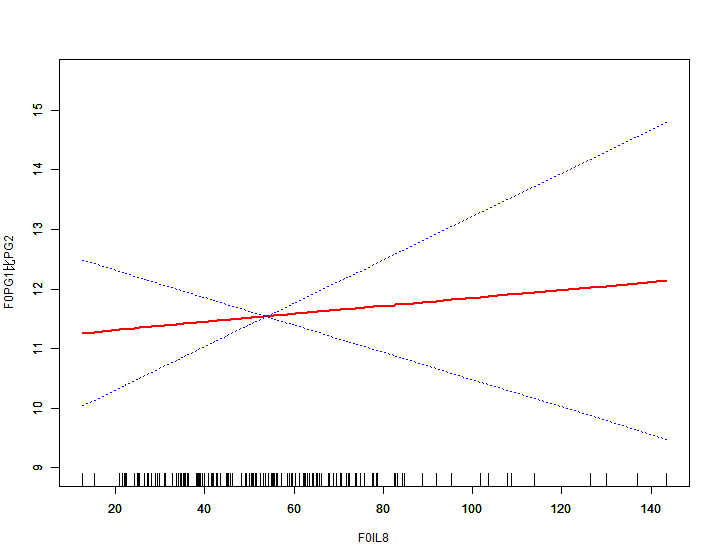

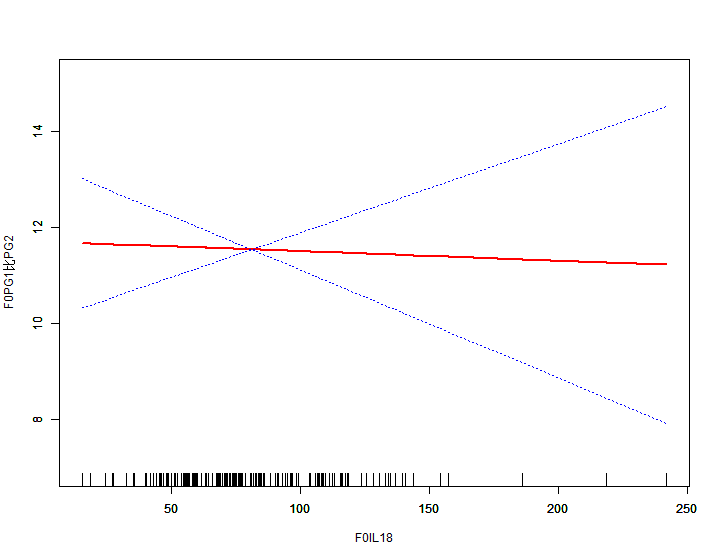

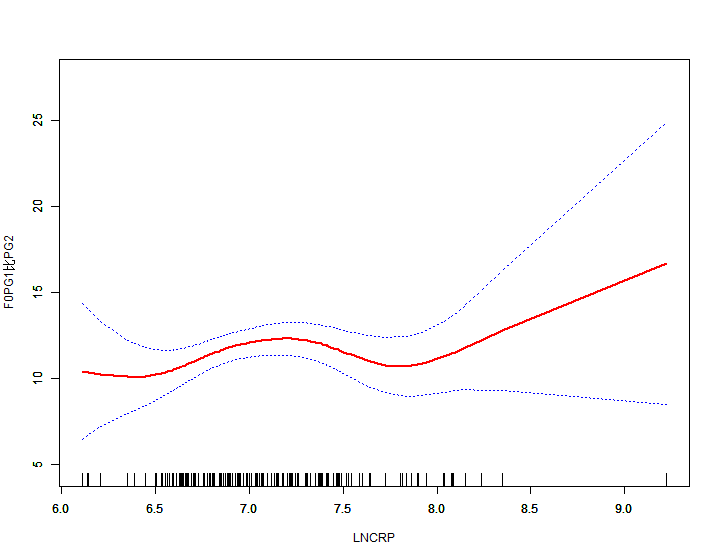

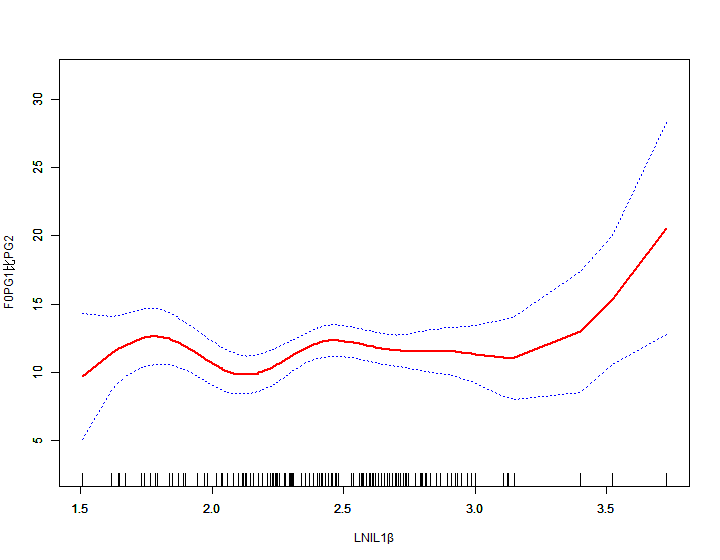


Figure S3 Inflammatory factors and PGR levels smooth curve fitting
